# A Potential Role of Natural Bioactive Compounds Found in Food in the Prevention of Idiopathic Parkinson’s Disease

**DOI:** 10.3390/nu17213376

**Published:** 2025-10-28

**Authors:** Sandro Huenchuguala, Juan Segura-Aguilar

**Affiliations:** 1 Escuela de Tecnología Médica, Facultad de Salud, Universidad Santo Tomás, Santiago 8370003, Chile; shuenchuguala@santotomas.cl; 2 Molecular & Clinical Pharmacology, Instituto de Ciencias Biomédicas (ICBM), Faculty of Medicine, University of Chile, Santiago 8380453, Chile

**Keywords:** neuroprotection, neurodegeneration, KEAP1/NRF2 pathway, aminochrome, dopamine, neuromelanin, natural compounds, omega-3, DT-diaphorase, glutathione transferase M2-2

## Abstract

Various clinical studies aimed at modifying the progression of idiopathic Parkinson’s disease have been unsuccessful. Similarly, several nutritional trials using bioactive compounds have shown positive effects for patients but have also failed to slow or reduce the disease’s progression. This repeated failure is likely because these studies ignore the extremely slow neurodegenerative process, which unfolds over many years. The molecular mechanism behind the loss of neuromelanin-containing dopaminergic neurons in the nigrostriatal system in idiopathic Parkinson’s disease remains unclear. This is a conceptual/theoretical review based mainly on mechanistic and preclinical evidence, with no direct clinical data. However, research suggests that aminochrome, an endogenous neurotoxin, may trigger the degeneration of these neurons through a single-neuron degeneration model. In this model, aminochrome selectively destroys individual neurons without spreading to neighboring cells. Aminochrome is produced during neuromelanin synthesis, a process that is normally harmless because protective enzymes like DT-diaphorase and glutathione transferase M2-2 neutralize aminochrome’s neurotoxic effects. Increasing the levels of these enzymes could offer neuroprotection. The KEAP1/NRF2 signaling pathway is critical for regulating antioxidant enzymes, such as DT-diaphorase and glutathione transferase M2-2. Importantly, specific bioactive compounds from food can activate this pathway, increasing the production of these protective enzymes. For instance, the omega-3 fatty acids eicosapentaenoic acid (EPA) and docosahexaenoic acid (DHA), along with astaxanthin—a compound present in cold-water fish like salmon—have been demonstrated to enhance enzyme expression. This connection leads to a compelling question: Could dietary interventions help prevent idiopathic Parkinson’s disease? Answering this will require further research.

## 1. Parkinson’s Disease

Parkinson’s disease is the second most common neurodegenerative disorder, characterized by the loss of dopamine-producing, neurons containing neuromelanin in the nigrostriatal system (NM-DA neurons). Most patients (70%) are diagnosed with idiopathic Parkinson’s disease, which primarily affects individuals aged 55–60 and older. Since 1967, the standard treatment has been L-dopa, which greatly improves motor function and helps patients maintain a near-normal life. However, after 4 to 6 years of treatment, side effects like dyskinesia often develop, significantly reducing patients’ quality of life. Despite extensive research, no new drugs have successfully slowed or stopped disease progression. Preclinical studies on compounds such as coenzyme Q, mitoquinone, urate, deferiprone, TCH346, and neurturin have shown promise, but these benefits have not carried over into clinical trials [1,2,3,4,5]. The failure of these trials has been attributed to flaws in trial design and the lack of reliable biomarkers [6,7,8].

However, we believe the failure of these clinical trials can be attributed to two key factors:

(i) The use of preclinical models that poorly replicate the disease process. These models rely on exogenous neurotoxins, which induce an extremely rapid and widespread degenerative process [9,10]. This sudden, aggressive degeneration seen in preclinical neurotoxin models sharply contrasts with the slow progression of idiopathic Parkinson’s disease, both before and after motor symptoms appear. The most common preclinical models for testing Parkinson’s drugs—1-methyl-4-phenyl-1,2,3,6-tetrahydropyridine (MPTP) and 6-hydroxydopamine—produce effects that are inconsistent with the natural course of the disease. For example, MPTP can induce severe parkinsonism in just three days in individuals exposed to contaminated drugs [11], whereas idiopathic Parkinson’s develops over many years, with neurodegeneration progressing gradually long before and after motor symptoms emerge. It is unlikely that a drug effective in these rapid, extreme neurotoxin models would translate to patients with idiopathic Parkinson’s, where degeneration occurs at a far slower pace.
(ii) The lack of a methodology capable of detecting subtle degenerative changes. Parkinson’s disease may follow a single-neuron degeneration model [12], consistent with its gradual progression before and after symptom onset. This raises the question of whether current tools, such as the Unified Parkinson’s Disease Rating Scale (UPDRS), are sensitive enough to measure these minute, incremental changes in neurodegeneration over time.

To this day, the exact triggers that initiate the loss of NM-DA neurons in idiopathic Parkinson’s disease remain unknown. However, scientists widely agree that multiple factors contribute to this process. These include mitochondrial dysfunction, oxidative stress, the formation of toxic alpha-synuclein aggregates, disruptions in both proteasomal and lysosomal protein-clearing systems, endoplasmic reticulum stress, and neuroinflammation [13,14,15,16,17,18,19,20].

## 2. Nutritional Trials in Parkinson’s Disease

The lack of new drugs to stop or slow the progression of idiopathic Parkinson’s disease has led researchers to investigate a possible link between diet and the risk of developing the illness. This degenerative process ultimately results in the loss of dopamine-producing neurons in the brain’s nigrostriatal system. Consequently, studies have examined the eating habits of different populations, as well as specific diets or nutrients that might offer a neuroprotective effect [21].

A study of 1053 patients with Parkinson’s disease found that supplements containing olive oil, wine, fresh fruit, unfried fish, coconut oil, fresh vegetables, herbs, spices, seeds, and nuts were linked to a slower progression of the disease [21]. In a separate study, researchers tracked the dietary habits of 131,368 healthy individuals to examine their relationship to the risk of developing Parkinson’s. Years later, 508 new cases were identified. The study concluded that a diet rich in vegetables, fruit, fish, legumes, nuts, and grains—coupled with moderate alcohol intake and low consumption of saturated fats—had a neuroprotective effect [22].

A pilot randomized trial was conducted to evaluate the role of a high-carbohydrate, low-fat diet versus a ketogenic diet in 47 patients with Parkinson’s disease. The study concluded that both diets improved the motor and non-motor symptoms of the disease [23]. A separate randomized clinical trial of the Mediterranean diet in 40 patients with Parkinson’s disease used the Montreal Cognitive Assessment Test to measure cognitive function at baseline and at the end of the study. This research found that the Mediterranean diet improved participants’ language skills, concentration, attention, working memory, and executive function [24]. Another study on the Mediterranean diet concluded that it had a positive effect on constipation and the gut microbiota of the patients in the study [25]. It has been suggested that the Mediterranean diet is associated with beneficial effects on gastrointestinal symptoms and improvements in global cognition [26].

A California study of 98 patients with Parkinson’s disease found that dietary changes can help reduce non-motor symptoms like constipation [27]. Separately, a study of 105 newly diagnosed Parkinson’s patients concluded that a Western diet increases the risk of developing the disease, whereas a healthy, traditional, and light diet was associated with a lower risk [28]. Various clinical trials have also explored the impact of polyunsaturated fatty acid (PUFA) supplementation. One 30-year study observing 8006 Japanese-American men noted a neuroprotective effect from these fats [29]. Another 6-year study of 5289 people who did not have Parkinson’s found that a high intake of unsaturated fatty acids lowered the risk of developing the disease [30].

A six-month study of 24 patients with Parkinson’s disease, conducted to determine the effect of docosahexaenoic acid (DHA), showed a positive effect in 75% of patients treated with DHA based on the Hamilton Rating Scale. However, no positive effect was observed when using the Hoehn-Yahr Scale and the Unified Parkinson’s Disease Rating Scale. The six-month DHA treatment reduced symptoms of depression [31]. In a separate 12-month, double-blind, placebo-controlled trial studying the effect of fish oil supplementation on 31 patients with Parkinson’s disease, the authors concluded that symptoms of depression decreased regardless of whether patients were also being treated with antidepressants [32].

A randomized, double-blind, placebo-controlled clinical trial was conducted with 60 patients with Parkinson’s disease and concluded that supplementation with omega-3 fatty acids and vitamin E had a positive effect on UPDRS scores [33]. A single-center, randomized, double-blind, placebo-controlled clinical trial involving 40 patients with Parkinson’s disease found that supplementation with Neuroaspis PLP10™ (ANIVA INTERNATIONAL 170 Syngrou Ave., 17671 Kallithea, Athens, Greece)—which contains omega-3 and omega-6 fatty acids, gamma-linolenic acid, vitamin A, and alpha- and gamma-tocopherol—slowed the progression of Parkinson’s disease as measured by the UPDRS [34]. Another randomized, double-blind, placebo-controlled clinical trial involving 40 patients with Parkinson’s disease concluded that omega-3 fatty acid supplementation, along with vitamin E taken for 12 months, significantly increased gene expression of peroxisome proliferator-activated receptor gamma, tumor necrosis factor alpha, and the low-density lipoprotein receptor. However, it had no effect on the expression of interleukin-1 and interleukin-8 [35].

A study tracked 135,916 men and women who showed no symptoms of Parkinson’s disease at the start of the research. Participants completed a dietary questionnaire, and over a follow-up period of 10 years for men and 16 years for women, 288 individuals were diagnosed with Parkinson’s. The study concluded that higher dietary caffeine intake was associated with a lower risk of developing the disease, suggesting that moderate caffeine consumption may have a neuroprotective effect [36]. In a separate clinical trial involving 60 Parkinson’s patients, researchers investigated whether curcumin could improve motor function, using standardized measures. The authors of that study found that curcumin did not lead to any improvement in clinical symptoms [37].

In a clinical study involving 46 Parkinson’s disease patients, participants were randomized to receive either a combination of probiotics and vitamin D or a placebo. The group receiving the active treatment showed a significant decrease in anxiety levels and gastrointestinal symptoms, assessed using the Beck Anxiety Inventory and the Gastrointestinal Symptom Rating Scale, respectively. Furthermore, significant improvements were noted in UPDRS subscales I, III, and IV, and in the total UPDRS score. In contrast, scores for UPDRS subscale II did not show a significant decrease [38].

## 3. The Effect of Pharmacological and Nutritional Interventions

Pharmacological and nutritional interventions share a common limitation: neither has been proven to stop or significantly slow the progression of Parkinson’s disease. Current drug therapies are only palliative, and clinical trials aimed at discovering new drugs that can modify the disease’s progression have consistently failed.

The most impactful pharmacological treatment for idiopathic Parkinson’s patients is L-dopa. Fifty-eight years after its introduction, it remains the most important medication. However, after four to five years of chronic use, severe side effects like dyskinesias often appear.

Similarly, nutritional interventions provide only partial benefits and fail to halt or slow the disease’s advancement. This raises the question of why these clinical studies and dietary approaches are unsuccessful in modifying the disease’s course.

In our view, the failure of both strategies stems from a fundamental misunderstanding of the disease’s degenerative process within the scientific community. This misunderstanding ignores the extremely slow nature of Parkinson’s. This misinterpretation hinders (i) the translation of successful preclinical studies into clinical trials for new disease-modifying drugs, and (ii) the effective implementation of nutritional interventions designed to alter progression. The problem is that disease progression unfolds over many years, while the interventions tested in unsuccessful clinical trials and nutritional studies are applied over a very short period. The progression of the disease after the onset of motor symptoms can last 10 to 20 years before death. Therefore, it has been proposed that the degenerative process of idiopathic Parkinson’s disease follows a single-neuron degeneration model [12].

## 4. Single-Neuron Degeneration

It has been proposed that the neurodegeneration affecting NM-DA neurons follows a single-neuron degeneration model [12]. This model explains why the degenerative process is so slow, taking years before motor symptoms emerge and continuing gradually throughout the disease. According to this model, the neurotoxin responsible for triggering mechanisms such as mitochondrial dysfunction, oxidative stress, neurotoxic alpha-synuclein oligomer formation, impaired proteasomal and lysosomal degradation, endoplasmic reticulum stress, and neuroinflammation originates within the neuron itself and does not spread to nearby cells.

Patients typically survive 10 to 20 years after diagnosis before succumbing to the condition. A recent study estimates that the total number of dopaminergic neurons in the substantia nigra (across both hemispheres) ranges from 800,000 to 1,000,000 [39]. For a patient who lives 15 years after motor symptoms begin—by which point 60% of NM-DA neurons are lost—this translates to a loss of 58 to 73 neurons per day. Such a slow progression can only occur if an endogenous neurotoxin selectively destroys neurons one at a time without affecting neighboring cells. Over time, the cumulative loss of these neurons eventually reaches a threshold where symptoms develop.

The single-neuron degeneration model is based on the fact that the endogenous neurotoxin, which triggers the degenerative process and the loss of dopaminergic neurons containing neuromelanin in the nigrostriatal system, is formed within these neurons and does not have an expansive character, affecting a single neuron. It has been proposed that the endogenous neurotoxin that is formed inside neurons and does not have an expansive character is aminochrome. Aminochrome is produced during neuromelanin synthesis where the catechol group of dopamine undergoes oxidation, generating three *ortho*-quinones in a sequential process: dopamine *ortho*-quinone, aminochrome, and 5,6-indolequinone. Among these, aminochrome is the most stable *ortho*-quinone and neurotoxicity that induces mitochondrial dysfunction, neurotoxic oligomer formation, oxidative stress, disruption of proteasomal and lysosomal protein degradation systems, endoplasmic reticulum stress, and neuroinflammation.

## 5. Why Is Not Neurotoxic During Neuromelanin Synthesis?

There seems to be a contradiction regarding the neurotoxic effects of aminochrome, which is produced during neuromelanin synthesis. Normally, neuromelanin synthesis is a harmless process—healthy elderly individuals often retain intact, NM-DA neurons at death [40,41,42]. This raises a key question: Why do healthy people not experience aminochrome’s neurotoxicity during neuromelanin synthesis? The answer lies in two critical enzymes that neutralize aminochrome’s harmful effects.

(i) DT-Diaphorase—DT-diaphorase (NAD(P)H: quinone oxydoreductase; NQO1; EC 1.6.99.2) is a distinct flavoenzyme that catalyzes the two-electron reduction of quinones to hydroquinones [43,44,45]. Inhibition of DT-diaphorase via siRNA has been demonstrated to trigger cell death in catecholaminergic cell cultures [46]. DT-diaphorase provides protection against: aminochrome-induced cell death, formation of neurotoxic α-synuclein oligomers, mitochondrial dysfunction, oxidative stress, autophagy and lysosomal dysfunction, disruption of cytoskeletal architecture [20,47,48,49,50,51,52,53,54].
(ii) Glutathione transferase M2-2—(EC 2.5.1.18). This enzyme catalyzes the conjugation of aminochrome with glutathione, forming 4-S-glutathionyl-5,6-dihydroxyindoline, a compound resistant to biological oxidizing agents such as superoxide, hydrogen peroxide, and dioxygen [55,56,57]. Glutathione transferase M2-2 also conjugates dopamine *ortho*-quinone (a precursor of aminochrome) to produce 5-glutathionyldopamine, which is typically metabolized into 5-cysteinyldopamine [58]. The detection of 5-cysteinyldopamine in human cerebrospinal fluid and neuromelanin suggests it is a stable end product, supporting its potential neuroprotective role. Notably, while glutathione transferase M2-2 is predominantly expressed in astrocytes, these cells secrete exosomes containing the enzyme, which then enter dopaminergic neurons and release the enzyme into their cytosol. This mechanism implies that astrocytes contribute to neuroprotection by boosting the defensive capacity of DT-diaphorase in NM-DA neurons [59,60,61,62].

**Figure 1:**
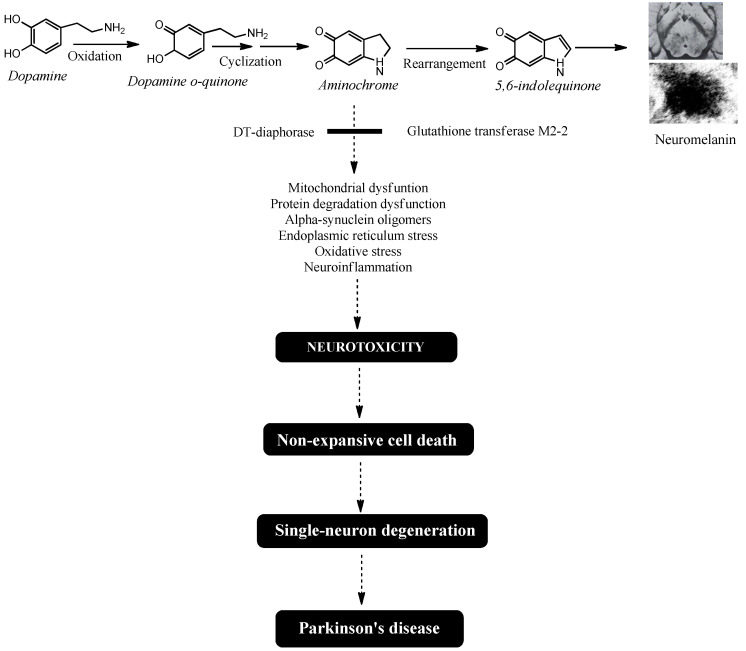
Aminochrome-induced single-neuron degeneration is a key process in Parkinson’s disease. The synthesis of neuromelanin within dopaminergic neurons requires the oxidation of dopamine’s catechol group. This reaction generates three *ortho*-quinones: dopamine o-quinone, aminochrome, and 5,6-indolequinone. Among these, aminochrome is the most stable and neurotoxic intermediate. It triggers a cascade of detrimental effects, including mitochondrial dysfunction, impaired protein degradation, the formation of neurotoxic alpha-synuclein oligomers, endoplasmic reticulum stress, oxidative stress, and neuroinflammation. Crucially, aminochrome’s neurotoxicity is highly focused, selectively damaging the neuron it forms in while sparing adjacent cells, resulting in single-neuron death. The slow, cumulative loss of these individual neurons over many years is what ultimately initiates the motor symptoms and drives the progression of idiopathic Parkinson’s disease.

The combined neuroprotective effects of DT-diaphorase and glutathione transferase M2-2 play a key role in preventing aminochrome-induced neurotoxicity during neuromelanin synthesis. However, decreased expression of these enzymes—along with excessive dopamine production and a resulting rise in aminochrome levels that overwhelms their protective capacity—may explain why NM-DA neurons are lost in Parkinson’s disease (Figure 1).

## 6. Bioactive Compounds in Food

Research suggests that plant-derived neuroprotective compounds could help prevent neurodegenerative diseases [63,64]. For example, nobiletin—a polymethoxylated flavone from *Citrus depressa* peel—has been shown to improve cognitive and motor deficits in preclinical Parkinson’s disease models [65]. Similarly, tangeretin, a citrus flavonoid found in the peel and other parts of *Citrus* L. plants, exhibits neuroprotective effects in MPTP− and MPP+-induced Parkinson’s models [66]. Other compounds, such as iridoids (geniposide, harpagoside, catalpol, and 10-O-trans-*p*-coumaroylcatalpol), have demonstrated neuroprotective activity in Parkinson’s models by boosting antioxidant enzymes (e.g., glutathione peroxidase and superoxide dismutase) and increasing tyrosine hydroxylase-positive neurons [67]. Polydatin, a natural compound in peanuts, grapes, and red wine, has shown neuroprotective effects by suppressing microglia activation and reducing pro-inflammatory factors in a lipopolysaccharide-induced Parkinson’s model [68]. Additionally, caffeic acid—a natural phenol in argan oil—has been found to protect dopaminergic neurons, enhance autophagy, and reduce alpha-synuclein aggregation in the substantia nigra of A53T alpha-synuclein transgenic models [69].

Chicoric acid, a polyphenol found in chicory and purple coneflower, has been shown to prevent MPTP-induced motor dysfunction, overactivation of glial cells, and the loss of dopaminergic neurons [70]. Morin, a flavonol present in wine and fruits, has been found to reduce motor dysfunction, protect dopaminergic neurons in the substantia nigra and striatum, and decrease astrocyte activation in an MPTP-induced mouse model. In primary cultures treated with MPP+, Morin demonstrated neuroprotective effects by lowering reactive oxygen species (ROS) production, preserving mitochondrial membrane potential, and inhibiting astroglial activation [71]. Wolfberry (the fruit of *Lycium barbarum* L.) has shown neuroprotective properties in multiple preclinical Parkinson’s disease models, including 6-hydroxydopamine-treated rats, MPTP-treated mice, and α-synuclein A53T mice. It helped alleviate motor deficits and prevented dopaminergic neuron loss by regulating iron metabolism [72]. In another study, extracts from *Vicia faba* L. sprouts increased dopamine levels in the striatum, improved motor function, reduced inflammatory markers, and lowered malondialdehyde levels in a rotenone-treated mouse model [73]. Additionally, research using MPTP-treated animal models and cell cultures found that the alkaloid *N*-methylene-(5,7,4-trihydroxy)-isoflavone, derived from *Sophora alopecuroides* L. fruits, reduced motor deficits, oxidative stress, neuroinflammation, and dopaminergic neuron loss in both the striatum and substantia nigra [74].

*Hericium erinaceus*, a medicinal mushroom, has demonstrated neuroprotective effects in neurodegenerative diseases like Parkinson’s. Its benefits are tied to boosting the production of neurotrophic factors [75,76]. Studies in rats have shown that *Cinnamomum osmophloeum* Kanehira extract increases dopamine and tyrosine hydroxylase levels while reducing alpha-synuclein buildup in the striatum. In the midbrain, it also enhances antioxidant enzymes like superoxide dismutase, catalase, and glutathione peroxidase [77]. Additionally, two neuroactive β-carbolines in coffee provide neuroprotective, antioxidant, and anti-inflammatory effects, potentially lowering Parkinson’s risk [78]. Another compound, nobiletin—a polymethoxylated flavone found in *Citrus depressa* peel—has been shown in animal models to improve both motor and cognitive deficits linked to Parkinson’s [65].

Resveratrol glucoside (also called polydatin), found in red wine, peanuts, and other foods, acts as a neuroprotectant in a preclinical model of lipopolysaccharide-induced Parkinson’s disease. It protects dopaminergic neurons from degeneration and improves motor dysfunction. Additionally, polydatin suppresses microglia activation and blocks the release of pro-inflammatory factors [68]. Caffeic acid, present in fruits, vegetables, coffee beans, and other dietary sources, reduces neurotoxicity caused by A53T alpha-synuclein overexpression in SH-SY5Y cells by activating the Nrf2/Bcl-2-mediated autophagy pathway [69]. Studies suggest that phytochemicals can prevent α-synuclein from forming neurotoxic oligomers and may even help break down existing aggregates [79]. Curcumin, a polyphenol in turmeric (*Curcuma longa*) used as a spice and food coloring, provides neuroprotection by modulating the brain-derived neurotrophic factor (BDNF) and PI3K/Akt signaling pathways [80]. Quercetin, abundant in apples, citrus fruits, onions, tea, and red wine, has been shown to inhibit alpha-synuclein aggregation into toxic oligomers [81]. Finally, L-theanine, found in green and black tea as well as certain mushrooms, exhibits neuroprotective effects in MPTP-treated SH-SY5Y cells. It boosts tyrosine hydroxylase-positive cells while decreasing alpha-synuclein clumping and Lewy body formation [82].

Omega-3 fatty acids, which are highly concentrated in salmon, have demonstrated neuroprotective effects in preclinical models of Parkinson’s disease [83,84,85,86]. These benefits are linked to multiple mechanisms, including: reducing endoplasmic reticulum stress, inhibiting microglial activation and the release of pro-inflammatory factors, decreasing mitochondrial dysfunction, promoting the expression of neurotrophic factors, maintaining calcium homeostasis and alpha-synuclein proteostasis [87]. In studies using unilaterally 6-hydroxydopamine-lesioned animals, treatment with fish oil for 50 days reduced neuronal loss in the substantia nigra pars compacta and their terminals in the striatum. The neuroprotection from fish oil was associated with fewer iNOS-immunoreactive cells and reduced microglial and astrocyte reactivity [88]. Additionally, omega-3 polyunsaturated fatty acids improved motor symptoms in 6-hydroxydopamine-treated animals, further confirming their neuroprotective role [89].

DHA, an omega-3 fatty acid, has been shown to restore tyrosine hydroxylase-positive neurons and decrease lipid peroxidation in rotenone-treated animals. It also boosts the production of antioxidant enzymes like catalase and superoxide dismutase [90]. In a rat model of 6-OHDA-induced Parkinson’s disease, DHA exhibited neuroprotective benefits by enhancing tyrosine hydroxylase levels and improving motor function, including gait and posture [91]. Additionally, DHA suppresses microgliosis and astrogliosis in both the substantia nigra and striatum in partial 6-OHDA lesion models, further supporting its neuroprotective effects [92].

The EPA has shown neuroprotective effects in differentiated human SH-SY5Y cells and primary mesencephalic cells exposed to MPP+ by countering the neurotoxin’s effects through the suppression of pro-inflammatory factor release [93]. Studies also indicate that EPA may help prevent Parkinson’s disease by reducing the neurotoxic effects of 6-hydroxydopamine in vitro. It helps restore mitochondrial function and boosts the expression of glial cell line-derived neurotrophic factor (GDNF) and brain-derived neurotrophic factor (BDNF), both of which are essential for neuronal survival, differentiation, and synapse formation [94]. Additionally, EPA has demonstrated protective effects in an MPTP-probenecid animal model, decreasing pro-inflammatory factor production and improving memory deficits [90]. A systematic review of 39 published studies further confirms the neuroprotective role of omega-3 fatty acids in Parkinson’s disease, noting improvements in behavior, pathological markers, and antioxidant, anti-inflammatory, and anti-apoptotic effects, along with higher omega-3 levels in the brain [95].

The dose of omega-3s used in different trials that showed a positive effect varied from study to study. Concentrations ranged from 1000 mg/day of omega-3s, 800 mg/day of DHA, and 290 mg/day of EPA, to 4140 mg/day and 810 mg/day [31,33,34,35]. The recommendation of daily intake dose of DHA and EPA to prevent diseases varies depending on the organization. The National Health and Medical Research Council recommends to prevent chronic illnesses with a total intake of omega-3 polyunsaturated fatty acids of 610 mg/day for men and 430 mg/day for women. The FAO and WHO recommend a total intake of DHA + EPA of 300 mg/day for women and 250 mg/day for men. However, daily intake of DHA + EPA is recommended by the American Heart Association to be 2000–4000 mg/day [96].

EPA and DHA are also found especially in fish, so a diet rich in fish that has a high content of these omega-3s can be a natural source in the prevention of Parkinson’s disease. Analysis of the percentage of DHA and EPA of the total lipids demonstrates that the herring contains 15% EPA and 22.6% DHA; Pollock roe contains 22% DHA and 18.8% EPA and salmon roe contains 17% DHA (85). The content of EPA in salmon and cultivated salmon is 5% and DHA is 10% and 9%, respectively [97]. The brown seaweed *Undaria pinnatifida*, used in the diet of the Asian population (Japan, China, Korea), contains 13% of its total lipids EPA. Almost 28% of the total lipids of flyingfish correspond to DHA. The freshwater fish *Cirrhinus mrigala*, also known as mrigal carp, is found in Pakistan, Bangladesh, northern India, and Nepal. 18% of its total lipids are DHA. Another river fish with high DHA content (18% of its total lipids) is *Catla catla*, which is found in Asian countries such as Thailand, Burma, India, Pakistan, Bangladesh and Nepal [80]. An analysis of EPA and DHA content in 39 fish used in the Indian diet showed that *C. catla*, *S. seenghala*, *T. ilisha* and *R. rita* had the highest content (6.8%, 4.4%, 2.9% and 3.8% EPA and 4.7%, 6.2%, 8.9% and 5% DHA of total lipids, respectively) [98]. The EPA and DHA contents in 100 g raw fillet of fish used in the diet in Chile were Peruvian morwong, Pacific pomfret, Chilean hake, Pacific sandperch, Chilean jack mackerel, Chub mackerel, Fine flounder, and the following were found to be 144, 23.6, 69, 141.9, 63.9, 45, and 32 mg EPA/100 g raw fillet, respectively. 295.8, 259, 148.9, 171.9, 279.8, 229.8, and 169.7 mg DHA/100 g raw fillet, respectively [99].

Astaxanthin (AST), a red dietary carotenoid found in foods such as salmon, krill, shrimp, crayfish, trout, yeast, and algae ([100,101]; Table 1), has neuroprotective, antioxidant, and anti-inflammatory properties. Its antioxidant effects come from its ability to increase the expression of DT-diaphorase and glutathione transferase M2-2 [102]. Notably, AST—along with DHA and EPA—activates the KEAP1/NRF2 signaling pathway, which boosts the production of these enzymes. These mechanisms are thought to play a key role in AST’s potential neuroprotective effects against Parkinson’s disease.

## 7. Natural Bioactive Compounds That Trigger Neuroprotection in NM-DA Neurons

The proposed neuroprotective role of DT-diaphorase and glutathione transferase M2-2 suggests that higher levels of these enzymes may be key in preventing neurotoxic effects on neuromelanin synthesis in idiopathic Parkinson’s disease. The increased expression of these antioxidant enzymes is controlled by the KEAP1/NRF2 signaling pathway, which includes DT-diaphorase and glutathione transferase M2-2 [103,104].

Bioactive compounds found in foods activate the KEAP1/NRF2 signaling pathway, inducing antioxidant enzymes. These bioactive compounds include nobiletin, tangeretin, geniposide, catalpol, polydatin/resveratrol glucoside, caffeic acid, chicoric acid, morin, wolfberry, hericium erinaceus, curcumin, and quercetin [105,106,107,108,109,110,111,112,113,114,115,116,117,118,119] (Table 2).

**Figure 2:**
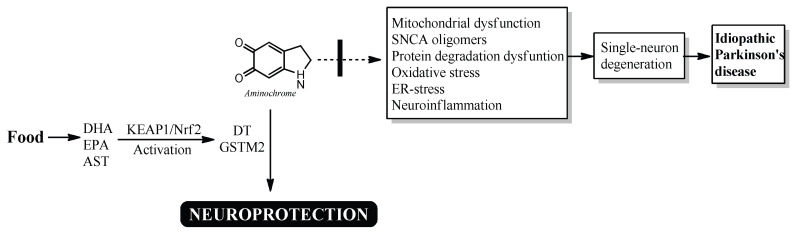
Possible neuroprotective mechanism of DHA, EPA and AST in idiopathic Parkinson’s disease. DHA, EPA, and AST activate the KEAP1/Nrf2 signaling pathway, increasing the expression of DT-diaphorase and glutathione transferase M2-2, which prevent the neurotoxic effects of aminochrome.

However, activation of the KEAP1/NRF2 signaling pathway by these bioactive compounds also includes increased expression of DT-diaphorase and glutathione transferase M2-2, although their expression was not determined in these studies (Figure 2).

## 8. Conclusions

Understanding the degenerative process that leads to the loss of NM-DA neurons is fundamental to the search for new drugs that can halt or slow the progression of Parkinson’s disease, as well as to identifying nutrients that may prevent or delay this neurodegeneration. In our view, the “single-neuron degeneration model” explains both the lack of success in clinical trials for new drugs and the difficulty in finding nutrients that help prevent and reduce the loss of these neurons.

Thus, increasing the expression of these enzymes could enhance neuroprotection in dopaminergic neurons when aminochrome is produced. The KEAP1/NRF2 signaling pathway plays a key role by activating the expression of antioxidant enzymes, including DT-diaphorase and glutathione transferase M2-2 [58,59]. There are no clinical studies to support the neuroprotective role of these enzymes in the loss of NM-DA neurons in idiopathic Parkinson’s disease. This is a conceptual/theoretical review based mainly on mechanistic and preclinical evidence, with no direct clinical data. Preclinical studies using aminochrome and a single-neuron degeneration model suggest that these enzymes may play a neuroprotective role in idiopathic Parkinson’s disease. While more research is needed to confirm this hypothesis, regularly incorporating foods rich in EPA and DHA into the diets of newly diagnosed Parkinson’s patients is likely not harmful.

Clinical studies on EPA and DHA have established their positive effects and determined safe dosage levels for when increasing dietary intake of these omega-3s is not feasible. Therefore, a key hypothesis for future research is that increasing the consumption of these bioactive-rich foods may enhance neuroprotection in dopaminergic neurons against aminochrome toxicity during neuromelanin synthesis.

## Figures and Tables

**Table 1:** Compounds and alpha-linolenic acid.

	Source	Amount of Total Lipids	Reference
Eicosapentaenoic acid (EPA)	Herring	15%	[80]
	Wild sardine	13.6% in muscle	[81]
	Pollock roe	18.8	[80]
	*Undaria pinnatifida*	13% of essential oil composition	[80]
	*Rhododendron sochadzeae*	2% of leaf extract	[80]
Docosahexaenoic acid (DHA)	Flyingfish	27.9%	[80]
	Herring	22.6%	[80]
	Pollock	22.2%	[80]
	Salmon roe	17.4%	[80]
	*Cirrhinus mrigata*	18.07 g/100 g muscle	[80]
	Catla catla	17.98 g/100 g muscle	[80]
	Jackalberry	4.54 g/100 g oil	[80]
Alpha-linolenic acid	Chia (*Salvia hispanica* L.) seed	64.04% of seed oil fatty acids	[80]
	*Trichosanthes kirilowii*	33.77–38.66% of seed oils	[80]
	Paprika *Capsicum annuum*	29.93% of fresh pericarp fatty acids in the Jaranda variety and 30.27% in the Jariza variety	[80]
	Sardine (*Sardina pilchardus*)	1.1	[81]
	*Linum usitatissimum*	1.1 to 65.2%	[80]
	Rapeseed oil	9.1%	[83]
	Olive oil	0.76%	[83]
	Flaxseed oil	53.4%	[83]
	Soybean oil	6.7%	[83]
	Corn oil	1.2%	[83]
	Walnut oil	10.4%	[83]
	Walnuts seed	9.0% of the total seed weight	[83]
	Flaxseed seed	22.8% of the total seed weight	[83]
	Hemp seed	10% of the total seed weight	[83]
Astaxanthin	Salmon		[98]
	krill		
	shrimp		
	crayfish		
	trout		
	yeast		
	algae		
	rainbow trout	0.172 mg/kg	
	Brook trout	0.103 mg/kg	[99]

**Table 2:** Bioactive compound that activate KEAP1/NRF2 signaling pathway.

Active Compound	Source	Reference
Nobiletin	*Citrus depressa* peel	[105]
Tangeretin	*Citrus* L. plants	[106]
Geniposide	*Gardenia jasminoides* Ellis	[107]
Catalpol	Fresh root of *Rehmannia*	[108]
Polydatin/Resveratrol glucoside	Grape, Polygonum cuspidatum	[109]
Caffeic acid	Coffee, apples, berries, plums	[110]
Chicoric acid	Chicory, purple coneflower	[111]
Morin	Wine and fruits	[112]
Wolfberry	*Lycium barbarum* L.	[113]
Hericium erinaceus	Fungus	[114]
Curcumin	Curcuma longa	[115]
Quercetin	Citrus fruits, onions, tea, and red wine	[116]
EPA	Wild sardine; herring, Pollock roe, undariapinnatifidia, Rhododendron, sochadzeae	[117]
DHA	Flyingfish, Herring, Pollock, salmon roe, cirrhinus mrigata, catla, catla, jackalberry	[118]
Astaxanthin	Salmon, krill, shrimp, crayfish, trout, yeast, and algae	[119]

## Data Availability

Not applicable.
